# Lack of keratinized mucosa increases peri-implantitis risk

**DOI:** 10.1038/s41432-023-00913-4

**Published:** 2023-08-03

**Authors:** Kelvin I. Afrashtehfar, Kyung Chul Oh, Carlos A. Jurado, Hyeonjong Lee

**Affiliations:** 1 Adjunct Research Professor, Department of Reconstructive Dentistry and Gerodontology, School of Dental Medicine, University of Bern, Bern, Switzerland; 2 Assistant Professor and Director of the Evidence-Based Practice Unit, Ajman University College of Dentistry, Ajman City, UAE; 3 Visiting Professor, Department of Prosthodontics, Yonsei University Dental Hospital, Seoul, South Korea; 4 Assistant Professor, Department of Prosthodontics, College of Dentistry, Yonsei University, Seoul, South Korea; 5 Associate Professor, Department of Prosthodontics, The University of Iowa College of Dentistry and Dental Clinics, Iowa City, IA USA; 6 Clinical Associate Professor, Department of Prosthodontics, College of Dentistry, Yonsei University, Seoul, South Korea

**Keywords:** Dental implants, Periodontics

## Abstract

**Design:**

A systematic appraisal and statistical aggregation of primary studies in humans.

**Data sources:**

The researchers utilized PubMed (Medline) and Scopus databases as the primary data sources for this study. They performed a comprehensive literature search based on free keywords and Medical Subject Heading (MeSH) terms to enhance the search accuracy. The database search was concluded on November 13, 2022. Furthermore, a meticulous examination of the references cited in the selected studies was conducted to identify additional relevant articles that could be incorporated into the analysis.

**Study selection:**

The systematic review focused on partially or fully edentulous patients receiving dental implants and aimed to determine if the lack of keratinized mucosa at the implant site increased the risk of peri-implantitis compared to patients with adequate keratinized mucosa. Human studies with a minimum of 100 implants, cross-sectional, cohort, or case-control designs, and a follow-up period of at least one year were included. Studies lacking a clear case definition or information on peri-implantitis and those that did not investigate keratinized mucosa as a risk indicator were excluded.

**Data extraction and synthesis:**

Two reviewers independently utilized a systematic review screening website (Rayyan, Qatar Computing Research Institute, Qatar Foundation) to select potential articles, and conflicts were resolved through discussion or consultation with a third reviewer. The data extraction process involved recording information from the included articles, such as study design, patient and implant numbers, prosthesis type (fixed or removable), follow-up duration, peri-implantitis case definition, prevalence at patient and implant levels, keratinized mucosa cutoff value, odds ratio (OR) of peri-implantitis considering keratinized mucosa, and conclusions on the potential effect of keratinized mucosa from each study. The Newcastle Ottawa scale (NOS) and a modified version of NOS were used, respectively, to assess the quality of cohort and cross-sectional studies. Studies scoring below 6 out of 9 points were classified as low quality. For the meta-analysis, the relationship between peri-implantitis and keratinized mucosa was evaluated using the odds ratio (OR) and standard error (SE). Heterogeneity was assessed through the Chi^2^ test and *I*^2^ index, determining whether a random-effects or fixed-effects model should be applied. Subgroup and cluster analyses were conducted based on specific criteria, and forest plots and funnel plots were generated to visualize results and identify potential study bias. Sensitivity analysis was performed to verify the robustness of the meta-analysis, with statistical significance set at *p* < 0.05. The Review Manager (RevMan) software facilitated data analysis.

The GRADE rating system was used to determine the level of evidence, considering factors such as bias risk, imprecision, inconsistency, indirectness, and publication bias. The certainty of the evidence was evaluated based on the overall outcomes of analyzed subgroups.

**Results:**

Twenty-two primary studies were identified, and a meta-analysis was conducted on 16 cross-sectional studies. The prevalence of peri-implantitis ranged from 6.68% to 62.3% at the patient level and from 4.5% to 58.1% at the implant level. The overall analysis revealed a significant association between the lack of keratinized mucosa and a higher prevalence of peri-implantitis (OR = 2.78, 95% CI 2.07–3.74, *p* < 0.00001). Subgroup analyses with a consistent case definition of peri-implantitis (MBL ≥ 2 mm) showed similar results (OR = 1.96, 95% CI 1.41–2.73, *p* < 0.0001). Studies focusing on fixed prostheses only demonstrated that the lack of keratinized mucosa was associated with an increased prevalence of peri-implantitis (OR = 2.82, 95% CI 1.85–4.28, *p* < 0.00001). Among patients under regular implant maintenance, the absence of keratinized mucosa significantly raised the occurrence of peri-implantitis (OR = 2.08, 95% CI 1.41–3.08, *p* = 0.0002). Studies adjusting for other variables also confirmed a higher risk of peri-implantitis with inadequate keratinized mucosa (OR = 3.68, 95% CI 2.32–5.82, *p* = 0.007). Although some publication bias was observed, the certainty of evidence based on the GRADE system was judged to be "moderate."

**Conclusions:**

The lack of keratinized mucosa increased the risk of peri-implantitis, emphasizing the need to consider it during dental implant placement. Inadequate data on patient-specific factors and the predominance of cross-sectional studies influenced the evidence quality (i.e., moderate). Future studies with consistent methodologies shall confirm these findings and identify additional risk indicators to improve implant dentistry practices.

## A Commentary on

**Mahardawi B, Jiaranuchart S, Damrongsirirat N et al.**

The lack of keratinized mucosa as a risk factor for peri-implantitis: a systematic review and meta-analysis. *Sci Rep* 2023; **13:** 3778.

**GRADE Rating:** 

## Commentary

Dental implants are widely used to replace missing teeth and restore oral function and aesthetics^1,2^. However, complications such as peri-implant mucositis and peri-implantitis can arise, posing risks to implant success^3,4^. Peri-implant mucositis involves inflammation without bone loss, while peri-implantitis entails progressive bone loss and mucosal inflammation^5^. These conditions can significantly impact peri-implant tissue health and compromise implant longevity^3^. Preventive measures and early risk assessment are crucial for managing peri-implant inflammations^6,7^ .Among various risk factors, the width of keratinized mucosa (KM) has been suggested as a potential indicator of peri-implantitis^8,9^. Studies have reported conflicting findings regarding the association between KM and peri-implantitis prevalence^10–12^, making it an area requiring further investigation. The reviewed meta-analysis by Mahardawi et al. aimed to analyze the available evidence and determine whether the absence of KM increased the risk of peri-implantitis. The synthesis also considered specific case definitions, restorative protocols, and maintenance conditions to mitigate potential confounding factors. In other words, the study intended to contribute to improving implant outcomes and patient care by clarifying the relationship between KM and peri-implantitis.

The review effectively addressed the research question, examining the impact of the absence of KM on the risk of peri-implantitis while considering relevant confounding factors. A thorough literature search identified 22 relevant articles, with 16 cross-sectional studies included in the meta-analysis. The overall analysis revealed a significant association between the lack of KM and a higher prevalence of peri-implantitis. Subgroup analyses consistently supported this finding, including case definition, fixed prostheses, regular maintenance, and adjustment for other variables. The quality assessment of the included studies indicated moderate to high quality. Although there might be some publication bias, the results overall suggest that the absence of KM is a risk factor that increases the prevalence of peri-implantitis. It is essential to consider this factor when placing dental implants. However, the review did not explicitly discuss the applicability of the findings or elaborate on potential biases in the review process. Moreover, a recent review and meta-analysis by Ravidà et al. ^13^. did not find a significant difference in peri-implant disease outcomes between sites with less than 2 mm or greater than or equal to 2 mm of KM width.

This systematic review possesses several notable strengths. Firstly, it includes a substantial number of studies, enhancing the reliability and robustness of the findings. Additionally, the review conducted multiple meta-analyses based on specific study features, such as case definition, prosthesis type, and maintenance frequency, thereby minimizing the impact of potential confounding factors and increasing the homogeneity of the results. Moreover, the review avoids fixating on a specific cut-off point for KM and instead considers various values, acknowledging the arbitrary nature of determining an optimal width.

Several limitations should be acknowledged. The predominant inclusion of cross-sectional studies limits the establishment of causality and may introduce bias. Thus, future longitudinal studies would provide more robust evidence in this regard. Furthermore, due to insufficient data, the review could not account for certain patient-related and site-specific factors, such as implant location, oral hygiene, time-in-function, and bone augmentation. The reliance on cross-sectional designs also introduces the possibility of deviation in outcomes based on the enrolled sample, potentially reducing the quality of evidence. Finally, although sensitivity analysis did not indicate significant changes, there is a slight asymmetry in the funnel plot, suggesting the presence of publication bias that should be considered when interpreting the results.

Future research on peri-implantitis should focus on longitudinal studies with standardized case definitions and analysis methods to establish stronger causal relationships. Additionally, exploring the impact of patient-related and site-specific factors, such as implant location, oral hygiene practices, time-in-function, and the influence of bone augmentation, will provide a more comprehensive understanding of peri-implantitis risk factors. Determining optimal cutoff points for KM width and its association with peri-implant tissue health could inform improved clinical practices in dental implantology.

To sum up, the lack of KM increased the risk of peri-implantitis. The review used a rigorous study selection process, data extraction, and quality assessment but could have addressed applicability and potential biases more explicitly. Additionally, the review did not reach high-quality evidence due to insufficient data on patient-specific factors and the predominance of cross-sectional studies. Future research should confirm these findings and identify additional risk indicators.

Practice points

- Adequate keratinized mucosa is essential for peri-implant tissue health. Clinicians should consider its presence and width during dental implant placement.
- Lack of keratinized mucosa increases the prevalence of peri-implantitis. Clinicians should be aware of this risk factor and consider it when assessing patients for implant placement.
